# Hypertension-Related Mortality in Aortic Dissection and Aneurysm: A Retrospective Observational Study

**DOI:** 10.7759/cureus.92769

**Published:** 2025-09-20

**Authors:** Meenakshi Yathindra, Aayushi Bhamat, Onkardeep Kaur, Tayyaba Mufti, Awad Mustafa, Navin Sampathkumar, Ajinkya Vijay Mahorkar

**Affiliations:** 1 Internal Medicine, Kasturba Medical College, Mangaluru, IND; 2 Internal Medicine, Godrej Memorial Hospital, Mumbai, IND; 3 Internal Medicine, Pandit Bhagwat Dayal Sharma Post Graduate Institute of Medical Sciences, Rohtak, IND; 4 Emergency Medicine, University Hospital Waterford, Waterford, IRL; 5 Internal Medicine, Baptist Memorial Hospital, Memphis, USA; 6 Medicine, Kasturba Medical College, Manipal, IND; 7 Cardiology, Avanti Institute of Cardiology, Nagpur, IND

**Keywords:** age-adjusted mortality rate, aortic aneurysm, aortic dissection, hypertension, retrospective study

## Abstract

Introduction: Hypertension is a major cause of cardiovascular event-related mortality, and its association with aortic aneurysm and dissection is being extensively studied.

Aim: To assess mortality trends and demographic disparities in hypertensive diseases with aortic dissection and aneurysm as a contributing cause.

Methodology: A retrospective observational study was conducted using the Centers for Disease Control and Prevention (CDC) Wide-ranging Online Data for Epidemiologic Research (WONDER) Multiple Cause of Death (MCD) database to assess mortality trends among individuals aged over 25 years in the United States from 1999 to 2020. Hypertension (ICD-10: I10-I15) was considered the underlying cause of death, with aortic dissection and aneurysm (ICD-10: I71.0) recorded as contributing causes. Data were stratified by gender, race, geographic region, and place of death. Age-adjusted mortality rates (AAMR) and annual percentage change (APC) were calculated.

Results: A total of 20,782 deaths were recorded, with a crude rate of 4.6 per million. The AAMR initially declined (-2.12% APC from 1999 to 2006) but increased significantly from 2006 to 2009 (+56.82% APC). It then decreased slightly from 2009 to 2020 (-0.42% APC). The highest mortality was observed in males (10,902, 52.5%), White individuals (16,551, 79.6%), metropolitan regions (17,426, 83.9%), and medical facilities (13,328, 64.13%). Temporal trends showed an increasing AAMR in both males (+57.22% APC from 2006 to 2009) and females (+56.32% APC from 2006 to 2009). A similar trend was observed during those years in African American individuals (APC +52.45%) and White individuals (APC +57.22%), indicating evolving disparities.

Conclusions: Mortality trends in hypertension with aortic dissection and aneurysm have shifted, with rising disparities in gender, race, geographic areas, and place of death. These findings underscore the need for targeted prevention strategies and improved healthcare access.

## Introduction

Hypertension, or high blood pressure, is a widespread chronic condition and a leading modifiable risk factor for cardiovascular morbidity and mortality. Globally, over 1.28 billion adults aged 30-79 years were estimated to have hypertension in 2021, with two-thirds living in low- and middle-income countries [1]. In the United States, data from the Centers for Disease Control and Prevention (CDC) reveal that between 1999 and 2020, nearly half (47.3%) of U.S. adults were hypertensive, yet only one in four had their condition under control [2]. Mortality associated with hypertensive diseases remains high, with increasing disparities based on age, sex, and race. Black adults are disproportionately affected, experiencing higher incidence, earlier onset, and greater mortality rates compared to White and Hispanic populations [3,4]. These disparities extend to regional and socioeconomic differences, with higher mortality reported in Southern states and rural areas.

Aortic aneurysm and dissection are acute, often fatal conditions of the aorta. An aneurysm represents a pathological dilation of the arterial wall, while a dissection involves a tear in the inner layer, creating a false lumen that can rupture or block blood flow to vital organs. These conditions are strongly associated with longstanding, poorly controlled hypertension, which induces stress and degenerative changes in the aortic wall [5]. Although relatively rare compared to other cardiovascular diseases, aortic aneurysms and dissections carry high mortality, especially when undiagnosed or untreated. Demographic analyses indicate higher rates in males, older individuals, and White populations, with recent data showing rising trends among African American individuals as well [6].

Pathophysiologically, sustained hypertension contributes to medial degeneration, loss of elastic fibers, and chronic inflammation in the aortic wall, predisposing individuals to both aneurysmal expansion and dissection. These mechanisms are particularly lethal when both conditions coexist, compounding the risk of rupture and sudden death. Prior research has emphasized hypertension as a standalone cause of death; however, fewer studies have explored its interplay with aortic aneurysm and dissection as contributing causes [7]. Most existing mortality analyses consider single-cause attribution, often overlooking the multifactorial nature of death. This creates a critical gap in understanding the full burden of hypertensive disease in complex cases involving vascular complications.

The CDC Multiple Cause of Death (MCD) database provides a unique resource to investigate mortality where hypertension is the primary cause and aortic pathology acts as a contributing factor. Analyzing such co-occurrence patterns offers valuable insights into demographic disparities, temporal trends, and healthcare access issues. This study aims to address the knowledge gap by evaluating mortality trends from 1999 to 2020, highlighting populations at increased risk, and informing integrated prevention strategies for hypertension and aortic disease.

This study aims to analyze mortality trends in hypertensive disease, where aortic dissection (AoD) and aneurysm are contributing causes of death, using the CDC Wide-ranging Online Data for Epidemiologic Research (WONDER) MCD database from 1999 to 2020.

The study stratifies data by gender, race, geographic area, and place of death to identify disparities in mortality patterns.

## Materials and methods

A retrospective original research study was conducted using the CDC WONDER MCD database. Data were extracted on April 21, 2025. As the database contains de-identified, publicly available data, the study was classified as non-human participant research and was therefore exempt from ethics committee approval.

The data extracted from the database covered the years 1999-2000 and included patients aged 25 years and above. Hypertension (I10-I15) was selected as the underlying cause of death, and aortic aneurysm and AoD were selected as multiple causes of death to assess the co-occurrence of these conditions. Demographic variables included sex (male and female) and race (American Indian or Alaska Native, Asian/Pacific Islander, Black or African American, and White) to account for disparities associated with these factors. Geographic variables were also considered, with categorization into metropolitan areas (large central metro, large fringe metro, medium metro, and small metro) and non-metropolitan areas (micropolitan and non-core). The place of death was categorized as a medical facility, home, or hospice. Mortality rates were age-adjusted per 1,000,000 population, using the 2000 U.S. standard population as the reference, to allow for accurate comparisons.

Descriptive data in the form of numbers and percentages were generated for the demographic and geographic variables using CDC WONDER MCD and the aforementioned criteria. Those not meeting the criteria were excluded from the study. Absolute numbers and percentages of mortality were derived from the database. Temporal trends in age-adjusted mortality rates (AAMRs) from 1999 to 2020 and the annual percentage change (APC) were derived using Joinpoint software version 5.3.00 (November 2024). These trends were assessed to identify statistically significant changes in mortality patterns across geographic and demographic variables.

## Results

From 1999 to 2020, the CDC MCD database recorded 20,782 deaths in the United States among individuals aged 25 years and older. Of these, cases with hypertensive disease (ICD-10: I10-I15) as the underlying cause of death and AoD or aneurysm (ICD-10: I71.0) listed as contributing causes were included in the study (*n* = 20,782). The crude mortality rate for hypertensive disease with AoD or aneurysm as a contributing cause was 4.6 per 1,000,000 population. Deaths not meeting these criteria were excluded.

Among the total deaths analyzed, males accounted for 10,902 (52.50%), while females accounted for 9,880 (47.50%). The mortality rate for hypertensive disease with AoD or aneurysm as a contributing cause was higher in males than in females, indicating a potential demographic disparity. Regarding racial distribution, the highest proportion of deaths occurred among White individuals (*n* = 16,551, 79.60%), followed by Black or African American individuals (*n* = 3,226, 15.50%), Asian or Pacific Islander individuals (*n *= 917, 4.40%), and American Indian or Alaska Native individuals (*n* = 88, 0.40%). The mortality burden was highest among White individuals, highlighting racial disparities in mortality trends related to hypertensive disease with AoD or aneurysm as contributing causes.

A majority of deaths occurred in metropolitan areas (*n* = 17,426, 83.90%), while non-metropolitan areas accounted for 3,356 (16.20%) deaths. Regarding the place of death, most deaths occurred in medical facilities (*n* = 13,328, 64.13%), followed by decedents' homes (*n* = 4,770, 22.95%), nursing homes or long-term care facilities (*n* = 1,635, 7.87%), and hospice facilities (*n* = 415, 2%).

Overall temporal trends

**Figure 1:**
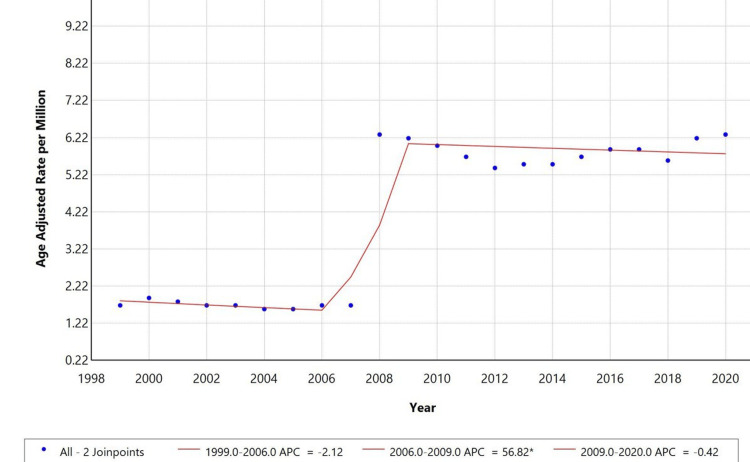
: Overall age-adjusted mortality rates among adults aged 25+ in the United States, 1999-2020. *Indicates that the annual percentage change (APC) is significantly different from zero at alpha = 0.05.

From 1999 to 2020, the AAMR for hypertensive disease with AoD and aneurysm as a contributing cause showed a decreasing phase, an increasing phase, and then a decreasing phase again. APC of -2.12% (*P* < 0.05) from 1999 to 2006. However, from 2006 to 2009, the AAMR increased significantly, with an APC of 56.82% (*P* < 0.05). A further decline was observed from 2009 to 2020, with an APC of -0.42% (*P* < 0.05). The shift suggests a notable change in mortality patterns over the past two decades; significant inflection points were observed in 2006-2009, indicating treatment advances, changes in disease prevalence, and public health interventions, as illustrated in Figure 1.

Gender-specific trends

**Figure 2:**
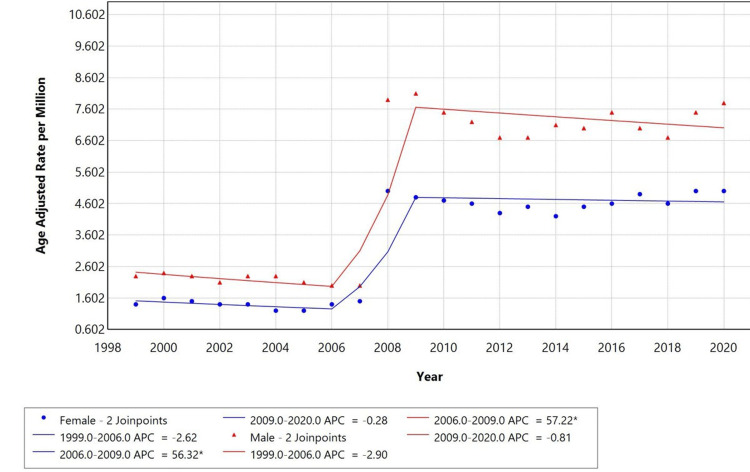
Trends in sex-stratified age-adjusted mortality rates among adults aged 25+ in the United States, 1999-2020. *Indicates that the annual percentage change (APC) is significantly different from zero at alpha = 0.05.

When stratified by gender, males had a higher AAMR compared to females. However, both groups exhibited similar trends, with a significant increase in mortality during 2006-2009, with an APC of 57.22% for males and 56.32% for females, and a decline from 1999-2006 and 2009-2020, as shown in Figure 2.

Race-specific trends

**Figure 3:**
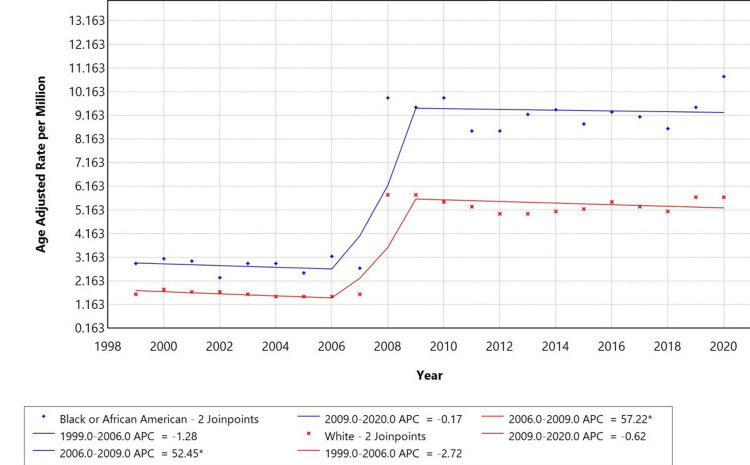
Trends in age-adjusted mortality rates stratified by race among adults aged 25+ years in the United States, 1999-2020. *Indicates that the annual percentage change (APC) is significantly different from zero at alpha = 0.05. Temporal trends for American Indian/Alaska Native and Asian Pacific Islander populations are not displayed due to data suppression for counts <10, limiting reliable trend analysis.

Racial disparities were observed in mortality trends. White individuals had the highest AAMR, followed by Black or African American and Asian or Pacific Islander individuals. The APC for White individuals was 57.22% from 2006 to 2009, while Black or African American individuals had an APC of 52.45% over the same period. Both groups showed a decline in AAMR from 1999 to 2006 and from 2009 to 2020. Trends for Asian or Pacific Islander and American Indian or Alaska Native individuals were not displayed due to data suppression for counts fewer than 10, limiting reliable trend analysis, as shown in Figure 3.

## Discussion

Using the CDC MCD database, this retrospective analysis evaluated mortality trends for hypertension (ICD-10: I10-I15) with aortic aneurysm and dissection (ICD-10: I71.0) as a contributing cause among persons aged 25 years and above in the United States from 1999 to 2020. Hypertensive diseases were identified as the primary cause of 20,782 deaths during this time, with aortic aneurysm and dissection being contributing factors. From 1999 to 2006, AAMR showed a varying trend: it declined for both sexes; from 2006 to 2009, it surged; and subsequently, it slightly decreased through 2020. Men accounted for 52.5% of deaths, White individuals for 79.6%, and metropolitan area residents for 83.9%. Most deaths occurred in medical facilities (64.13%), followed by the deceased’s homes (22.95%).

Aortic aneurysms and dissections (I71.0) are largely caused by chronic hypertensive diseases (I10-I15), which put the aortic wall under constant biomechanical stress. Persistent high blood pressure compromises aortic wall integrity and increases the risk of aneurysmal dilation and consequent dissection or rupture by accelerating medial degeneration, promoting smooth muscle death, and upsetting elastin and collagen networks [8]. Furthermore, hypertension creates a pro-inflammatory environment that regulates matrix metalloproteinases (mostly MMP-2 and MMP-9), causing extracellular matrix degradation, a fundamental component of aneurysm pathogenesis [9]. Strong epidemiological overlap is suggested by large population-based studies by Landenhed et al., which show that hypertension exists in up to 70%-80% of patients with thoracic AoD and in almost 50% of those with abdominal aortic aneurysms (AAAs) [10]. Chronic hypertension can act as a trigger for intimal tears when dissecting aortic segments. Elevated blood pressure and vascular wall weakening have a synergistic effect that greatly increases the likelihood of catastrophic events, such as aortic rupture or dissection, which helps explain the higher mortality when both conditions coexist [11].

With hypertensive diseases (I10-I15) listed as the underlying cause and aortic aneurysm or dissection (I71.0) as a contributing cause of death, this study found an overall AAMR of 4.6 per million among adults aged 25 years and older from 1999 to 2020. The striking increase in mortality between 2006 and 2009, with APC exceeding +56% to 57% in both sexes, was followed by a modest but steady drop in rates through 2020. Although earlier studies have separately examined mortality from aortic aneurysms and hypertension, very few have looked at their co-occurrence in death records at a national level. Related studies have, however, also recorded similar death changes. Landenhed et al. noted, for example, that the mortality risk for aortic events in patients with hypertension remained much higher even with improved vascular care, underscoring the ongoing lethality of combined vascular pathology [10]. In the United Kingdom, Howard et al. also recorded stable to slightly increasing AoD deaths; they emphasized that improved imaging and diagnosis increased case ascertainment without appreciably affecting mortality. According to their research, the primary risk factor was poorly controlled blood pressure; many individuals died before ever reaching the hospital. This emphasizes the need for early blood pressure management to prevent such potentially fatal events [12]. A study's sex-stratified trends indicate that older adults (≥85 years), men, non-Hispanic Black individuals, and women experienced the steepest increases in mortality during this period [13]. Contrary to reports implying increasing rates, another study by Melvinsdottir et al. [14], based on Icelandic registry data, shows that the incidence of acute thoracic aortic dissection (ATAD) in Iceland stayed constant at 2.53 per 100,000/year from 1992 to 2013. Over 50% of patients, particularly those with Stanford type A dissection, died within 30 days, indicating that mortality was still high. Reflecting developments in surgical and endovascular management, encouragingly, 30-day mortality dropped and five-year survival improved over time [14].

There are several plausible explanations for the patterns observed in our study. Transitions in ICD-10 coding enforcement and increasing physician awareness of documenting hypertension and vascular comorbidities on death certificates may also correspond to the mortality surge from 2006 to 2009. Public health policies promoting hypertension control, such as the Million Hearts® Initiative, launched in 2012, sought to reduce cardiovascular mortality in high-risk populations by implementing evidence-based interventions aimed at controlling blood pressure and cholesterol, quitting smoking, and using aspirin [15]. Furthermore, Clough et al. claimed that increasing use of high-resolution MRI and CT angiography could have helped to detect AoDs post-mortem, thus improving the reporting of this disorder as a contributing cause. Faster aortic expansion is also correlated with high stroke volume, velocity, and helical flow, which, in type B AoD, can result in poor long-term outcomes and a higher risk of death. These findings suggest that reducing mortality depends primarily on early imaging and monitoring [16]. These initiatives and increased adherence to the U.S. Preventive Services Task Force recommendations for one-time AAA screening in older male smokers most certainly helped lower AAA-related death and rupture rates. Nevertheless, given that screening has not been linked to a decrease in all-cause mortality, the plateau and modest drop in mortality observed after 2009 may also reflect improvements in surgical management, imaging, and general cardiovascular risk control strategies [17].

White individuals accounted for the highest number of deaths (16,551, 79.6%) compared to other racial groups, followed by Black or African American individuals (3,226, 15.5%). Men accounted for a higher percentage of deaths (10,902, 52.5%) compared to women (9,880, 47.5%), clearly showing gender disparities. This is consistent with earlier studies showing that men usually have higher AAMRs from aortic aneurysm and hypertension-related complications, possibly due to biological differences in vascular elasticity and lifestyle risk factors. AAAs affect 9% of people over the age of 65, with White males being the most common (5:1 ratio), and the risk of growth and rupture increases dramatically above 5 cm. Common comorbidities are cardiac diseases and hypertension. AAAs are rare, but they are frequently degenerative. Gender and comorbidity patterns are evident in aorto-iliac occlusive disease, with patients who have hypertension or diabetes experiencing poorer outcomes [18]. Furthermore, research has demonstrated that Black individuals have disproportionately higher mortality rates from hypertensive cardiovascular diseases. This is attributed to their earlier onset of hypertension, reduced access to quality healthcare, and greater exposure to social determinants of health, such as poverty and discrimination [4]. Goyal et al. found that non-Hispanic White individuals had the highest mortality, followed by American Indian/Alaska Native and African American individuals; Hispanic and Asian/Pacific Islander individuals had lower AAA rates [19]. In contrast, the lower percentages among Asian or Pacific Islander (4.4%) and American Indian or Alaska Native populations (0.4%) in our study may reflect either true epidemiological differences or underreporting due to disparities in diagnosis and healthcare access. These demographic trends highlight the intersection of systemic obstacles, healthcare disparities, and biological susceptibility that contribute to adverse outcomes.

Geographic variation influences health outcomes, particularly cardiovascular mortality. Our findings showed that metropolitan areas accounted for 83.9% of all deaths, with large central metro areas contributing the highest proportion (31.7%). Nevertheless, when population size and healthcare access are taken into consideration, people living in non-metropolitan areas, especially non-core rural communities (6.8%), are likely to experience disproportionately higher AAMRs. Reduced availability of cardiovascular specialists, delayed diagnosis resulting from limited diagnostic facilities (e.g., lack of advanced imaging like CT angiography), lower rates of routine screening, and a higher burden of unmanaged hypertension and smoking in rural populations could all help to explain this trend. Further aggravating these differences are environmental exposures, shortages in the healthcare workforce, and social determinants, including income level and education [20]. With limited healthcare access, workforce shortages, and a higher burden of risk factors, including smoking, obesity, and poverty, rural women experience higher mortality. Geographic location is therefore an important factor in outcomes of women’s cardiovascular disease [21].

Using CT imaging in adults aged ≥55, Hinojosa et al. conducted a multicenter study spanning nine academic institutions in four major metropolitan areas of Mexico, which evaluated undetected AAA. With similar rates across areas, the overall AAA frequency was 3.08%, suggesting a consistent geographic distribution. Small differences in detection rates among centers highlight the need for standardized screening, particularly in metropolitan areas where diagnosing tools are more accessible [22]. Boyle et al. noted that although peri-operative mortality following AAA repair has dropped globally, notable geographical differences still exist, as shown by endovascular aneurysm repair (EVAR) usage rates ranging from 35% in Hungary to 81% in the United States, highlighting disparities in healthcare infrastructure and access to modern surgical techniques [23]. Reflecting differences in access to vascular care, imaging, and emergency services, Goyal et al. found that non-metropolitan areas consistently showed higher AAMRs (50.7 per 1,000,000) than metropolitan areas (39.2 per 1,000,000), even if overall AAA mortality from 1999 to 2020 showed a general decline. Delayed diagnosis and treatment among rural populations could worsen outcomes [19]. While surgical rates dropped notably in São Paulo and Minas Gerais, Franco et al. noted that AAA mortality in Brazil increased in many areas, particularly in the South, Southeast, and Central-West. The Northeast displayed limited operations and increasing mortality. The fact that many of the most affected areas were rural or less urbanized emphasizes the need for focused surgical access and public health interventions [24]. Similarly, using Global Burden of Disease data, Roth et al. found significant regional differences in hypertension and heart disease mortality, with higher rates in rural and economically deprived areas [25]. Singh and Siahpush underlined that, compared to metropolitan areas from 1969 to 2009, the rural areas in the United States have seen consistently higher CVD death rates. Poor Black populations were most affected by this disparity, and limited progress in rural heart disease and stroke outcomes further contributed to the rural-urban mortality gap [26]. These patterns highlight how urgently focused public health campaigns and resource allocation are needed to control aneurysms and lower hypertension in high-risk rural communities.

The AAMR for deaths with hypertensive diseases (I10-I15) as the underlying cause and aortic aneurysm/dissection (I71.0) initially declined between 1999 and 2006, then sharply increased between 2006 and 2009, followed by a moderate decline. Reflecting ongoing differences in cardiovascular health, men and White individuals experienced disproportionately higher mortality. These patterns coincide with earlier studies suggesting that, especially in areas with limited access to preventive care and vascular imaging services, both aortic aneurysms and hypertensive heart disease remain underdiagnosed or poorly managed in specific demographic and regional groups [27]. From a public health standpoint, these results emphasize how urgently early detection techniques, such as combining blood pressure screening and aneurysm surveillance, have to be strengthened. Targeted health education campaigns focusing on medication adherence, smoking cessation, and dietary changes may also help reduce modifiable risk factors for hypertension and aneurysmal disease. Geographic variation in mortality also highlights systemic inequalities in healthcare access and specialist availability, supporting policy-level interventions to increase cardiovascular and vascular surgical capacity in non-metropolitan areas [28].

This retrospective study is subject to the limitations of death certificate data, including potential coding errors, underreporting, and lack of clinical details such as smoking status, diabetes, or chronic kidney disease. The ICD-10 codes do not distinguish between Stanford type A and B dissections or thoracic and abdominal aneurysms, limiting disease-specific analysis. Data suppression for small subgroups also limited trend assessment in certain populations. As an observational study, our findings show associations rather than causation.

Future research should investigate how race-, sex-, and region-specific risk factor profiles interact with care delivery paths to impact outcomes. Furthermore, longitudinal cohort studies and implementation research are needed to evaluate community-based screening programs, telemedicine follow-up for hypertension control, and the incorporation of vascular risk assessment tools into routine care. To address the rising burden of cardiovascular mortality from hypertensive disease and related vascular complications, policymakers and healthcare systems must prioritize equitable resource allocation and culturally tailored preventive strategies.

## Conclusions

This study highlights the initial decline in mortality among hypertensive patients with aortic dissection and aneurysm between 1999 and 2006. This initial decrease was followed by a significant increase in mortality trends from 2006 to 2009. After 2009, AAMR continued to decline modestly. The highest burden was observed in males, White individuals, medical facilities, and metropolitan areas, revealing evolving disparities.

These results emphasize the need for studies and future research to address gender, racial, and geographic disparities. Integrated cardiovascular care emphasizing early detection and treatment, implementation of early cardiovascular risk assessment, public health efforts to improve healthcare access, reallocation of resources to high-risk rural areas, and enhancement of preventive strategies, screening, and surveillance protocols are needed to reduce mortality.
